# Obesity is associated with decreased lung compliance and hypercapnia during robotic assisted surgery

**DOI:** 10.1007/s10877-016-9831-y

**Published:** 2016-01-28

**Authors:** Dana Rodica Tomescu, Mihai Popescu, Simona Olimpia Dima, Nicolae Bacalbașa, Șerban Bubenek-Turconi

**Affiliations:** 1 Department of Anesthesiology and Critical Care III, Fundeni Clinical Institute, 258 Fundeni Street, 2nd District, Bucharest, 022328 Romania; 2 “Carol Davila” University of Medicine and Pharmacy, Bucharest, Romania; 3 “Dan Setlacec” Center of Gastrointestinal Disease and Liver Transplantation, Fundeni Clinical Institute, Bucharest, Romania; 4 Department of Anesthesiology and Critical Care, “C.C. Iliescu” Institute for Cardiovascular Disease, Bucharest, Romania

**Keywords:** Robotic assisted surgery, Hypercapnia, Mechanical ventilation, Lung compliance, Respiratory physiology

## Abstract

Robotic assisted surgery (RAS) represents a great challenge for anesthesiology due to the increased intraabdomial pressures required for surgical optimal approach. The changes in lung physiology are difficult to predict and require fast decision making in order to prevent altered gas exchange. The aim of this study was to document the combined effect of patient physical status, medical history and intraoperative position during RAS on lung physiology and to determine perioperative risk factors for hypercapnia. We prospectively analyzed 62 patients who underwent elective RAS. Age, co-morbidities and body mass index (BMI) were recorded before surgery. Ventilatory parameters and arterial blood gas analysis were determined before induction of anesthesia, after tracheal intubation and on an hourly basis until the end of surgery. In RAS, the induction of pneumoperitoneum was associated with a significant decrease in lung compliance from a mean of 42.5–26.7 ml cm H_2_O^−1^ (*p* = 0.001) and an increase in plateau pressure from a mean of 16.1 mmHg to a mean of 23.6 mmHg (*p* = 0.001). Obesity, demonstrated by a BMI over 30, significantly correlates with a decrease in lung compliance after induction of anesthesia (*p* = 0.001). A significant higher increase in arterial CO_2_ tension was registered in patients undergoing RAS in steep Trendelenburg position (*p* = 0.05), but no significant changes in end-tidal CO_2_ were recorded. A higher arterial to end-tidal CO_2_ tension gradient was observed in patients with a BMI > 30 (*p* < 0.001). In conclusion, patients’ physical status, especially obesity, represents the main risk factor for decreased lung compliance during RAS and patient positioning in either Trendelenburg or steep Trendelenburg during surgery has limited effects on respiratory physiology.

## Introduction

The revolution introduced in the field of general surgery by performing laparoscopic procedures is tremendous. Robotic assisted surgery (RAS) represents the latest innovation in minimally invasive surgery and its use has became widely spread in all subspecialties of general abdominal surgery including gynecologic procedures, colorectal surgery, gastric surgery and hepatobiliary surgery [1].

The advantages of RAS on patient postoperative recovery are well known and consist of reduced analgesic requirements and a shorter time to discharge [2–4]. On the other hand, robotic technologies allow the surgeon to have a three-dimensional view of the operating field, an increased instrumental degree of freedom and surgeon motion filtration [5].

For the anesthesiologist RAS represents a challenge. In order to facilitate surgical movement, the patient is placed in either Trendelenburg (T) or steep Trendelenburg (sT) position for the duration of surgery, and this, combined with carbon dioxide (CO_2_) induced pneumoperitoneum is likely to cause significant changes in respiratory physiology. The large amounts of gas insufflated are absorbed via the peritoneal surface and lead to hypercapnia and respiratory acidosis if the CO_2_ cannot be excreted by increased minute ventilation.

## Materials and methods

The ethical approval for the present study was provided by the Ethical Committee of Fundeni Clinical Institute, Bucharest, Romania. The aim of this study was to investigate the combined effect of patient physical status, medical history and intraoperative position during RAS on respiratory physiology. Additionally, we sought to determine a predictive model for patients prone to develop hypercapnia in the intraoperative period due to CO_2_ pneumoperitoneum and to establish the correlation between end-tidal CO_2_ (EtCO_2_) and arterial CO_2_ pressure (PaCO_2_) in this setting.

### Patient inclusion

In the present study we prospectively analyzed 62 patients who underwent elective abdominal RAS in the Department of General Surgery and Liver Transplantation at Fundeni Clinical Institute during a 4 months period (October 2012–January 2013). Exclusion criteria consisted of: age under 18 years and conversion of RAS to laparotomy. All surgeries were performed under general anesthesia. Patients were classified according to the American Society of Anesthesiologists (ASA) physical status as ASA II or III.

### Anesthetic management

After arrival in the operating room, standard monitoring was applied to all patients: electrocardiography, pulse oximetry, non-invasive arterial blood pressure and body temperature. 5 mg of intramuscular midazolam were administrated as premedication 30 min prior to surgery. Induction of anesthesia was performed using propofol (1–2 mg kg^−1^) and fentanyl (2–4 µg kg^−1^). Succinylcholine (1–1.5 mg kg^−1^) was used in order to facilitate tracheal intubation. Anesthesia was maintained with Sevoflurane adjusted to maintain a minimal alveolar concentration between 1 and 1.5. Fentanyl was used for intraoperative analgesia and was administered as required by the clinician’s judgment. Neuromuscular blockade was obtained using atracurium with a loading dose of 0.5 mg kg^−1^ followed by 0.1 mg kg^−1^ at 20–45 min intervals. No neuromuscular blockade reversal agents were used. The degree of muscle relaxation was monitored by train of four. The lungs were ventilated using a Primus Ventilator (Dräger Medical^®^, Lübeck, Germany) in a volume controlled mode with an oxygen/air mixture of 0.5. Ventilator settings were: tidal volume of 6–8 ml kg^−1^, inspiratory/expiratory ratio 1:2 and 2.0 l min^−1^ of inspiratory fresh gas flow. Positive end-expiratory pressure was not used. Respiratory rate was adjusted to maintain an EtCO_2_ pressure of 36 ± 4 mmHg.

After tracheal intubation a 22 G arterial catheter (Arrow International Inc^®^ Reading, PA, USA) was inserted percutaneously into the radial artery in order to invasively monitor arterial blood pressure and to collect blood samples for arterial blood gases analysis. The right internal jugular vein was cannulated using a three way central venous catheter for fluid replacement and central venous pressure monitoring. Body temperature was maintained by infusing worm fluids (Hotline Blood and fluid warming System—Level 1^®^, Smith Medical, St. Paul, MN, USA) and using a convective warming system (Equator—surface warming system^®^, Smith Medical, St. Paul, MN, USA).

Patient positioning was applied in either T or sT in order to facilitate surgical approach. The surgeries were performed using a da Vinci Robot Surgical System (Intuitive Surgical^®^, Sunnydale, CA, USA) by a transperitoneal approach. Pneumoperitoneum was obtained by CO_2_ insufflation and was automatically maintained at 12–14 mmHg. At the end of the procedure each patient returned to the supine position, the pneumoperitoneum released and the patient was awakened either in the operating room or in the Postanesthesia Care Unit according to the clinicians’ decision.

### Collected data

Patient demographic data, co-morbidities, chronic medical treatment, history of smoking and COPD and intended surgical procedure were collected by the attending anesthesiologist before surgery. Preoperative spirometry was performed in all patients who were actively smoking before surgery or who had a prior diagnosis of chronic pulmonary disease in order to identify those suffering of COPD or chronic asthma. Arterial blood samples were obtained for blood gases analysis (ABL 800 Radiometer Medical APS^®^, Brǿnshǿj, Denmark) after tracheal intubation, after induction of pneumoperitoneum, on an hourly basis thereafter and at the end of surgery. Ventilator parameters, including tidal volume (TV), respiratory rate (RR), minute-ventilation (MV), plateau airway pressure (Pplat), lung compliance (after performing an inspiratory hold maneuver) and EtCO_2_ were noted at the same time as arterial blood gas analyses. The arterial to EtCO_2_ gradient was calculated as the arithmetic difference between the measured arterial oxygen pressure (PaO_2_) and the mean EtCO_2_ during the minute before obtaining the arterial blood sample. Lung compliance (C) was defined as a pulmonary compliance during periods without gas flow, such as during an inspiratory pause. The normal lung compliance was considered at values of 0.05 l cm H_2_O^−1^. Decreased C after induction of pneumoperitoneum was considered a fall in C of more than 30 %.

### Statistical analysis

Statistical analyses were performed using SPSS 19.0 (SPSS Inc^®^, Chicago, IL, USA). Data are presented as mean ± standard deviation of the mean, median (min, max) otherwise percentage. Data distribution was examined in order to insure the proper statistical examination. Categorical variables were analyzed with Chi square test and quantitative data were analyzed with independent samples *t* test. Mann–Whitney test was used when the analyzed data did not follow a normal distribution. Repeated measurements were analyzed using an ANOVA test. In order to analyze the combined effect of patient positioning and body mass index (BMI) a two-way ANOVA test was applied. All *p* values are two-tailed and a *p* value of <0.005 was considered statistically significant.

## Results

**Fig. 1:**
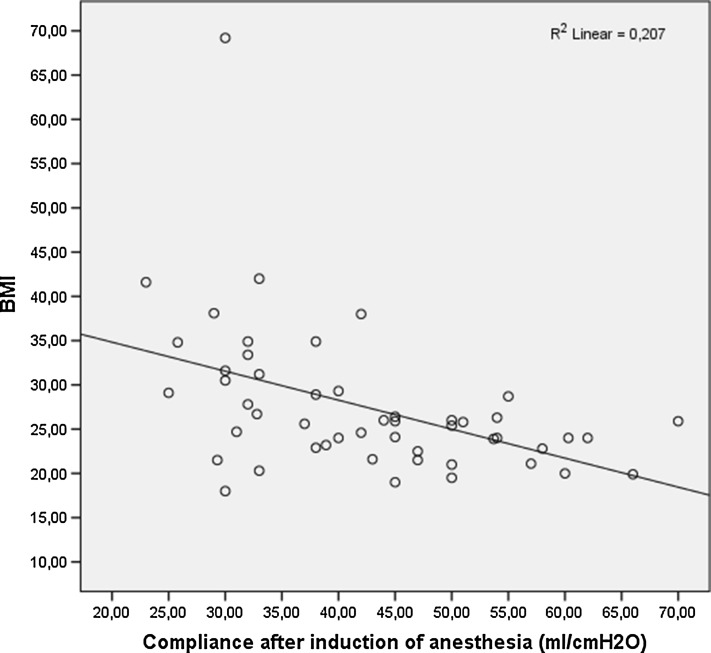
Correlation between lung compliance after induction of anesthesia and BMI

Fifty patients were enrolled in this study out of the total of 62 screened. Two patients were excluded because of incomplete data and ten surgeries were converted from RAS to laparotomy. Of the 50 patients remaining 62 % (n = 31) were female and 38 % (n = 19) were male. Surgical indications consisted of colectomy in 19 patients (38.0 %), gastrectomy in 14 patients (28 %), hysterectomy in 8 patients (16.0 %). Twenty-three patients (46 %) had a BMI under 25 and approximately half of patients were obese: 15 patients (30 %) had a BMI between 25.1 and 30 and 12 patients (24 %) had a BMI over 30.1. The mean duration of surgery was 251.7 ± 87.7 min and that of pneumoperitoneum was 200.5 ± 76 min. We found no significant differences in duration of surgery between patients with a BMI of under 25 and those with a BMI above 25 (*p* = 0.288) or between patients with a BMI under 25 and those with a BMI above 30 (*p* = 0.291). We also found no significant statistical differences between type of surgery and patient BMI. The mean age in the study group was 56.8 years. 42 % of patients (n = 21) were actively smoking at the time of surgery. A history of smoking (cigarettes) was positive in 21 patients (42 %) and 7 patients (14 %) had a history of pulmonary disease (either chronic asthma or COPD). None of 7 patients had an acute exacerbation of chronic pulmonary disease prior to surgery, as evidenced by clinical symptoms or by spirometry. Lung compliance after induction of anesthesia (Ci) was 42 ± 11.5 ml cm H_2_O^−1^. Correlations between demographic data Ci are shown in Table 1. Only BMI over 30 significantly correlated with a decrease in lung compliance after induction of anesthesia (*p* = 0.001; Fig. 1).

**Table 1:** Factors associated with low lung compliance after induction of anesthesia

Factor	Mean value/percentage	*p* value
Age (years)	56.8 ± 16.7	0.404
Sex		
Male	38 % (n = 19)	0.225
Female	62 % (n = 31)	
BMI	27.7 ± 8.32	
<25	46 % (n = 23)	
25–30	30 % (n = 15)	0.078
>30	24 % (n = 12)	0.001*
Previous abdominal surgery	34 % (n = 17)	0.212
Cardiovascular pathology	42 % (n = 21)	0.707
Pulmonary pathology	14 % (n = 7)	0.278
History of smoking	42 % (n = 21)	0.894
ASA II/III	5/45	0.554

*BMI* body mass index, *Pplat* plateau inspiratory pressure, *ASA* American Society of Anesthesiologist physical status

Statistically significant result is indicated in bold

* *p* < 0.05

T position was applied in 52 % of patients (n = 26). The mean duration of surgery was 262 ± 76 min in the T group and 240 ± 98 min in the sT group (*p* = 0.299). The mean duration of pneumoperitoneum 200 ± 61 min in the T group and 200 ± 90 min in the sT group (*p* = 0.490). Six patients with a BMI > 30.1 were included in the T group and five patients with a BMI > 30.1 were included in the sT group.

**Fig. 2:**
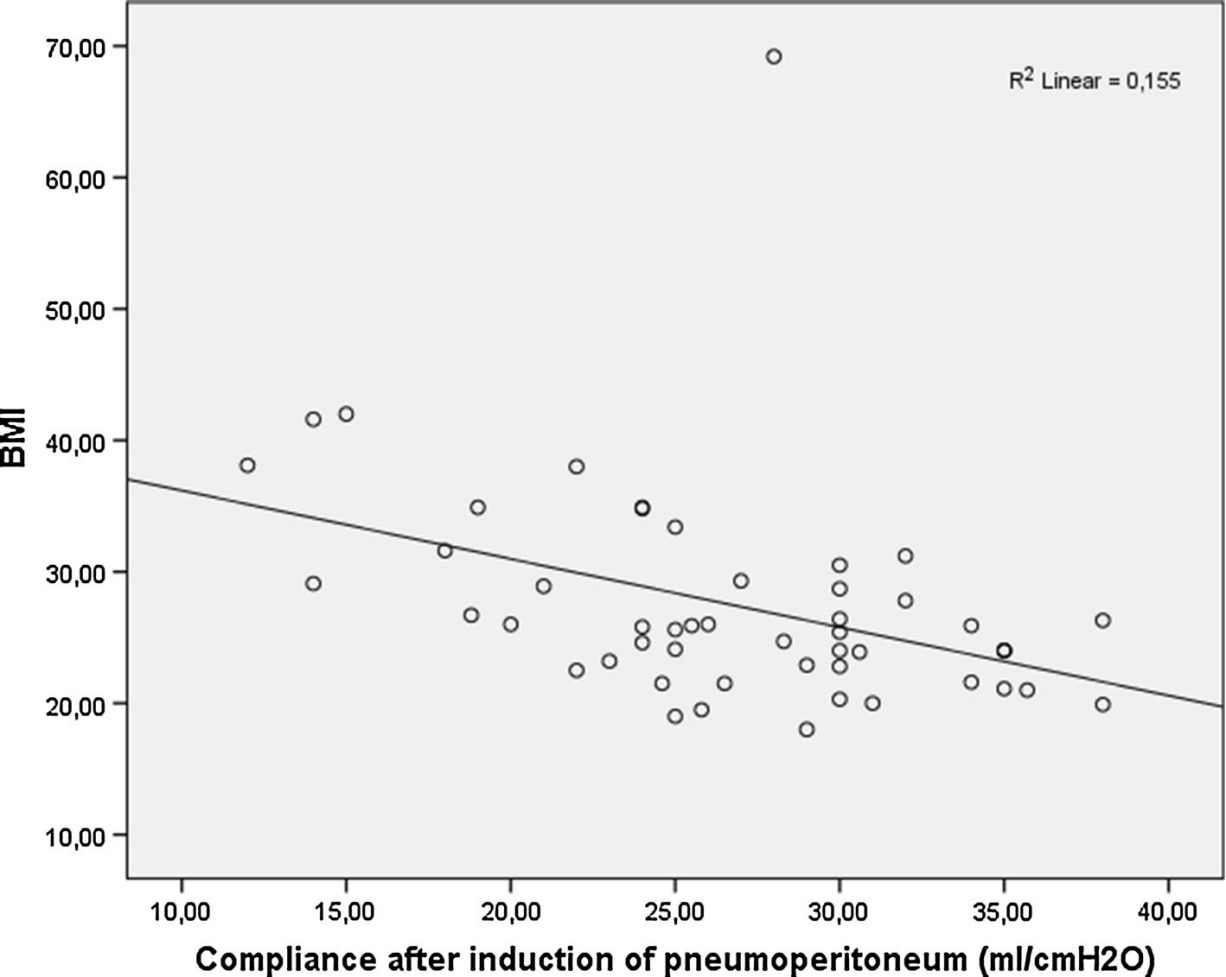
Correlation between lung compliance after induction of pneumoperitoneum and BMI

After induction of pneumoperitoneum the mean C decreased by 61 % (from a mean of 42.5–26.7 ml cm H_2_O^−1^)—Fig. 2. The Pplat increased from a mean of 16.1 mmHg to a mean of 23.6 mmHg. Differences in ventilatory parameters between T and sT position are presented in Table 2. There were no significant correlations between T and sT in regard to Pplat, MV or C. Differences in ventilatory parameters between patients with a BMI < 25, patients with a BMI between 25.1 and 30 and patients with a BMI > 30 are presented in Table 3. We observed significantly higher Pplat in obese patients after induction of anesthesia (*p* = 0.000) and pneumoperitoneum (*p* = 0.000). No combined effect of obesity and patient positioning on Pplat was observed. C was significantly lower in obese patients after induction of anesthesia (*p* = 0.000) and pneumoperitoneum (*p* = 0.000). The combined effect of patient positioning and BMI on C did not reached statistical significance.

**Table 2:** Mean (SD) measured variables in the intraoperative period compared with Trendelenburg (T) or steep Trendelenburg (sT) position

Parameter	Trendelenburg position (n = 26 patients)	Steep Trendelenburg position (n = 24 patients)
PaCO_2_–EtCO_2_ (mmHg)		
T0	6.1 (4.6)	9.7 (6.1)
T1	7.70 (6.2)	12.7 (5.8)*
T2	10.8 (6.3)	13.6 (5.0)*
Tf	10.2 (7.3)	11.8 (6.8)
Pplat (mmHg)		
T0	16.6 (4.8)	15.6 (5.5)
T1	24.3 (5.3)	22.7 (2.7)
T2	24.0 (6.3)	23.0 (6.1)
Tf	19.4 (5.2)	21.3 (6.8)
Minute ventilation (MV) (ml min^−1^)		
T0	6.8 (0.8)	6.6 (1.6)
T1	8.2 (1.2)	8.4 (2.0)
T2	8.4 (1.4)	8.5 (2.5)
Tf	7.6 (1.4)	8.5 (1.7)
Respiratory rate (RR)		
T0	12.2 (0.6)	12.7 (1.9)
T1	15.0 (2.4)	16.7 (2.1)
T2	15.5 (2.6)*	17.5 (1.7)
Tf	13.8 (2.1)*	16.5 (2.5)
Lung compliance (C)		
T0	44.6 (12.5)	40.2 (10.2)
T1	27.0 (6.8)	26.5 (5.7)
T2	29.5 (9.1)	26.5 (7.5)
Tf	37.0 (10.6)	31.3 (8.6)

T0 refers to time after induction of anesthesia, T1 after induction of pneumoperitoneum, T2 to the median intraoperative time and Tf—end of surgery

* *p* < 0.005

**Table 3:** Mean (SD) measured variables in the intraoperative period compared between patients with a BMI < 25, BMI 25.1–30 and BMI > 30.1 and effect of combined patient positioning and BMI on respiratory parameters

Parameter	BMI < 25	BMI 25–30	BMI > 30	*p* value	Combined Trendelenburg and BMI effect (*p* value)
PaCO_2_–EtCO_2_					
T0	8.0	3.9	4.1	0.973	0.705
T1	4.7	6.9	6.7	0.042	0.010
T2	5.4	5.1	3.9	0.301	0.925
Tf	6.0	4.1	9.7	0.021	0.866
Pplat (mmHg)					
T0	14.1	15.4	22.2	0.000	0.515
T1	20.5	24.2	30.4	0.000	0.403
T2	20.2	24.6	30.0	0.000	0.188
Tf	17.6	20.4	26.6	0.000	0.030
MV (ml min^−1^)					
T0	6.45	6.70	7.35	0.957	0.253
T1	8.30	8.43	8.40	0.941	0.411
T2	8.13	9.42	9.23	0.014	0.048
Tf	7.59	8.54	8.67	0.188	0.262
Lung compliance (C)					
T0	45.9	43.6	32.2	0.000	0.290
T1	28.8	26.1	22.6	0.000	0.360
T2	32.3	25.7	21.6	0.000	0.094
Tf	37.8	32.5	28.2	0.000	0.156

T0 refers to time after induction of anesthesia, T1 after induction of pneumoperitoneum, T2 to the median intraoperative time and Tf—end of surgery

sT position correlated with a higher PaCO_2_ value after induction of pneumoperitoneum (*p* = 0.048) and after 1 h (*p* = 0.05). In order to maintain normocapnia a higher RR was required for patients in sT for the remaining duration of surgery. The EtCO_2_ remained approximately constant throughout surgery and no differences were observed between patients in T and sT position. A higher PaCO_2_–EtCO_2_ gradient was observed for patients in sT position (*p* = 0.033) and in obese patients after induction of pneumoperitoneum (*p* = 0.042) and at the end of surgery (*p* = 0.021). Patients with a BMI > 30.1 and positioned in sT had a significantly higher PaCO_2_–EtCO_2_ gradient after induction of pneumoperitoneum (*p* = 0.010).

## Discussion

BMI represents the main risk factor for decreased lung compliance after induction of anesthesia and insufflation of pneumoperitoneum. Our data suggest that there are no differences in Pplat, C or MV in patients undergoing RAS in regard to T or sT positioning. Obesity, defined as a BMI > 30.1, significantly associated with decreased lung compliance and increased Pplat throughout surgery.

Altered lung compliance has been suggested by Andersson [6] who argued in their study on seven patients that a CO_2_ pneumoperitoneum of 11–13 mmHg increases the volume of atelectasis by displacing the diaphragm cranially. In the present study, C decreased by approximately 15.8 ml cm H_2_O^−1^ and Pplat increased by 7.5 mmHg. A study by Suh [7] found decreased dynamic C and increased Pplat during gynecologic laparoscopic surgery independent of T or sT positioning of the patient. They found the same degree of increase in Pplat, but a much lower decrease in C.

Obesity represents an important determinant of respiratory mechanics in patients undergoing surgery under general anesthesia by decreased functional residual capacity, decreased compliance and increased resistance of the total respiratory system [8]. This is in accordance with our study and that of Kim [9] and Sprung [10] where patient BMI and insufflation of pneumoperitoneum had the most important influence on respiratory mechanics. In the present study, the decrease was 61 % compared with a 30 % decrease in the study performed by Kim [9]. This may be explained by the differences in patients’ physical status (ASA classification) between the two studies.

Our findings suggest that pneumoperitoneum alter respiratory mechanics probably by pushing the diaphragm upwards to such an extent that further changes, like patient positioning, cannot affect in a significant manner respiratory dynamics. All studies referred to enrolled patients undergoing elective laparoscopic surgery in ASA I–II patients without pulmonary pathology. Although we tried to evaluate the impact of known pulmonary disease, especially COPD and asthma, for further alteration of pulmonary function, our data lack statistically relevance. This is mainly due to the small number of patients with pulmonary disease included in our study. Further data are required in order to better assess the impact of pulmonary pathologies on lung function during RAS.

Changes in lung function after induction of pneumoperitoneum and patient positioning remained approximately constant throughout surgery. After removal of pneumoperitoneum and return to supine position the ventilatory variables did not returned to baseline. This is in disagreement with Rauh [11] who stated that 5 min after deflation, all values returned to baseline levels.

PaCO_2_ increased after CO_2_ insufflation into a much greater extent in patients in sT position, while EtCO_2_ remained relatively constant. This lead to the significantly higher PaCO_2_–EtCO_2_ gradient observed in patients in sT. We also observed a higher gradient for obese patients that were operated on in sT position. This is in discordance with published studies [12–14]. Our hypothesis is that our results reflect the relative large amount gastric surgeries included in our study in which the surgical tools may press directly on the diaphragm and limit ventilatory mechanics. In their study, von Ungern-Sternberg [15] observed that vital capacity decreased to a much greater extent in patients with a BMI > 30, than in non-obese patients during surgery. Moreover, they demonstrated that spirometry parameters decreased significantly more after lower abdominal laparotomy then after breast cancer surgery.

The effect of T position on hemodynamic variables has been extensively studied [16] and specific hemodynamic changes have been observed in patients undergoing laparoscopic surgery in T position [17]. In their study, Meininger [18] found no significant cardiovascular depression in patients undergoing RAS during T position, while Falabella [19] observed that mean arterial pressure and vascular resistance increase during surgery in sT position. Kalmar [20] studied the effects of sT and CO_2_ pneumoperitoneum on cardiovascular, cerebrovascular and respiratory homeostasis during RAS. They observed that Pplat increased and C decreased after induction of pneumoperitoneum. After reinstitution of the supine position, Pplat returned to slightly above baseline levels and a residual loss of C was registered. Their results are in accordance to ours and may be explained by basal atelectasis that may develop during surgery.

Tomescu [16] found that pressure gradient increases with age. We could not reach such a conclusion in our study group. This is probably due to a much wider range of surgical procedures and older patients included in our study group. Further data are required to investigate age-related respiratory physiological changes in RAS.

von Ungern-Sternberg [21] demonstrated in their study that the use of epidural analgesia correlated with a much lower decrease in vital capacity after midline laparotomy for gynecological procedures. The use of epidural analgesia was not considered in our study, but it may be feasible in RAS in order to reduce opioid-based analgesia and minimize respiratory depression during the early postoperative period. Also in their study, von Ungern-Sternberg reported that vital capacity reduction was more pronounced in obese patients. Their results are in accordance with our observation.

In conclusion RAS has a marked effect on the respiratory function during surgery and pneumoperitoneum. Our predictive model of patients prone to develop changes that dramatically affect respiratory physiology consists of obese patients that have borderline or decreased lung compliance and high plateau pressures after induction of anesthesia regardless of expected surgical positioning. Further studies are required in order to better understand the effect of both pulmonary pathology and age-related respiratory physiological changes in patients undergoing RAS.
